# Kaposi’s Sarcoma-Associated Herpesvirus-Encoded Viral IL-6 (vIL-6) Enhances Immunoglobulin Class-Switch Recombination

**DOI:** 10.3389/fmicb.2018.03119

**Published:** 2018-12-18

**Authors:** Santas A. Rosario, Gabriel E. Santiago, Enrique A. Mesri, Ramiro E. Verdun

**Affiliations:** ^1^Sylvester Comprehensive Cancer Center, University of Miami Miller School of Medicine, Miami, FL, United States; ^2^Department of Microbiology & Immunology, University of Miami Miller School of Medicine, Miami, FL, United States; ^3^Sheila and David Fuente Graduate Program in Cancer Biology, University of Miami Miller School of Medicine, Miami, FL, United States; ^4^Division of Hematology, Department of Medicine, University of Miami Miller School of Medicine, Miami, FL, United States; ^5^Miami Center for AIDS Research, University of Miami Miller School of Medicine, Miami, FL, United States; ^6^Geriatric Research, Education, and Clinical Center, Miami VA Healthcare System, Miami, FL, United States

**Keywords:** Kaposi’s sarcoma-associated herpesvirus, viral IL-6, adaptive immunity, class-switch recombination, heavy chain constant region, microhomology, classical non-homologous end joining, activation-induced cytidine deaminase

## Abstract

Kaposi’s sarcoma-associated herpesvirus (KSHV) is an oncogenic gamma-herpesvirus that causes AIDS-associated Kaposi sarcoma (KS) and several lymphoproliferative disorders. During the humoral immune response antigen-activated mature B cells acquire functional diversification by immunoglobulin heavy chain (IgH) class-switch recombination (CSR). CSR is initiated by activation-induced cytidine deaminase (AID) which targets highly repetitive switch (S)-regions to mediate DNA double-stranded breaks (DSBs) in the IgH locus facilitating intramolecular recombination. Here we show that in the context of cytokine stimulation, CSR is enhanced in murine B cells exposed only to replication-competent KSHV in an environment of KSHV infection, which coincided with elevated AID transcripts. Using murine splenic B cells and the mouse lymphoma CH12F3-2 CSR system, we identified that vIL-6, but not murine IL-6, increased class-switching, which correlated with upregulated AID expression. Together, these data suggest a regulatory role for KSHV vIL-6 in functionally modulating B cell biology by promoting CSR, which may in part explain how KSHV infection influences humoral immunity and affect KSHV pathogenesis.

## Introduction

Kaposi’s sarcoma-associated herpes virus (KSHV) is an oncogenic gamma-herpesvirus that is the underlying cause of Kaposi sarcoma (KS), an AIDS-associated angiogenic cancer of endothelial cell origin, and several lymphoproliferative malignancies namely, primary effusion lymphoma (PEL), multicentric Castleman Disease (MCD), and germinotropic lymphoproliferative disorder (GLD) (Du et al., 2002; Mesri et al., 2010; Bhavsar et al., 2017). The majority of KSHV-related diseases manifest in immunocompromised individuals, primarily in settings of coinfection with HIV and EBV, and other opportunistic pathogens (Mesri et al., 2010; Thakker and Verma, 2016). A theory of how KSHV promotes oncogenesis is via paracrine neoplasia (Mesri et al., 2010). KSHV passively replicates its episomal DNA and subverts immune detection by latently infecting the majority of cells (Mesri et al., 2010; Bekerman et al., 2013; Host et al., 2017). However, a small subset of cells within the population undergo lytic replication and produce various inflammatory, angiogenic and proliferative factors that activate their cognate receptors via paracrine signaling on latently and non-infected cells (Bais et al., 1998; Cesarman et al., 2000; Pati et al., 2001; Mesri et al., 2010). Thus, KSHV-related pathogenesis is a consequence of immune dysfunction, HIV co-infection, and perturbation of signaling mechanisms that are exploited by the dual phases of the KSHV life cycle.

To successfully establish lifelong persistence within a host, KSHV employs several strategies, including mimicry of host cytokines, to modulate the immune response and usurp cellular signaling pathways (Moore et al., 1996; Chen et al., 2009; Host et al., 2017). One crucial cytokine is vIL-6, a viral homolog to human IL-6 (hIL-6) and mouse IL-6 (mIL-6). vIL-6 plays a pivotal role in the pathogenesis of all KSHV-associated malignancies (Aoki et al., 2001). Its secretion is detected from KSHV-infected B cells where it is expressed during lytic replication and at decreased levels during latency (Giffin et al., 2015) promoting angiogenesis, and stimulating cellular proliferation, survival, IL-6 production, and cell movement (Burger et al., 1998; Mullberg et al., 2000; Aoki et al., 2001; Chen et al., 2009; Giffin et al., 2015). Importantly, whereas IL-6 only stimulates gp130 via direct binding to IL6R (gp80), vIL-6 can bypass binding of gp80 and directly activate gp130 to initiate downstream signaling cascades (Wan et al., 1999; Mullberg et al., 2000). In contrast to IL-6 which is actively secreted from the cell, vIL-6 is maintained within the endoplasmic reticulum (ER) via interactions with calnexin and VKORC1v2, imparting vIL-6 with a longer secretory half-life than IL-6 (Meads and Medveczky, 2004; Chen et al., 2009, 2012, 2014). vIL-6 also targets the JAK/STAT pathway by inducing STAT1 and STAT3 activation (Molden et al., 1997).

One component of humoral immunity is class-switch recombination (CSR), an intramolecular recombination mechanism that changes the biological effector function of an antibody while preserving its antigen-specificity (Dedeoglu et al., 2004). During CSR, B cells exchange the IgM heavy chain constant (C)-region for a downstream C-region, changing the isotype from IgM to another such as IgG, IgE, or IgA (Stavnezer et al., 2008). CSR is initiated by activation-induced cytidine deaminase (AID) which is part of the ‘apolipoprotein B mRNA editing enzyme, catalytic polypeptide-like’ (APOBEC) family of cytidine deaminases and is only found at high concentrations in GC B cells (Muramatsu et al., 2000; Crouch et al., 2007). AID is recruited to highly repetitive deoxycytidine (dC)-rich sequences on the immunoglobulin (Ig) heavy chain locus (IGH) called switch (S)-regions which precede C-regions. At the donor S-region, Sμ, and downstream acceptor S-region, AID deaminates dC to deoxyuridines (dU) creating G:U mismatches. This initiates non-canonical DNA excision repair pathways that generate DNA double-strand breaks (DSBs) in both S-regions which then recombine via end-joining mechanisms (Stavnezer et al., 2008).

It is postulated that B cells contribute to KSHV-related pathogenesis by functioning as reservoirs for the virus, serving as vessels for viral dissemination and cytokine secretion (Lee et al., 2012; Knowlton et al., 2014). While it is recognized that KSHV infection of B cells is associated with the development of lymphoproliferative disorders and KS, there is a paucity of literature regarding how KSHV modulates humoral immunity, during acute KSHV infection. It is evident that KSHV affects various aspects of humoral immunity. Antibody titers against the virus are variable and are observed for years before the onset of KS, indicating that the humoral arm of the immune system is incapable of effectively clearing the virus (Gao et al., 1996; Biggar et al., 2003; Kimball et al., 2004; Kumar et al., 2013; Olp et al., 2015, 2016). Further, it is hypothesized that KSHV favors a Th2 over a Th1-mediated immune response (Douglas et al., 2010). Notably, KSHV has been shown to upregulate AID expression in B cells (Bekerman et al., 2013) and modify Ig light chain specificity (Totonchy et al., 2018).

Hereby, we employ two available murine models of CSR to demonstrate here that B cells exposed to KSHV enhanced the efficiency of CSR. Using a murine B-cell lymphoma line (CH12F3) and primary splenocytes, we show that increased CSR is likely mediated by AID and occurs only in the context of co-stimulation with specific cytokines/ inflammatory environments. Furthermore, we show that viral IL-6 (vIL-6), but not murine IL-6, augmented CSR. Our data suggest a novel function for vIL-6 in enhancing Ig CSR in B cells and may provide insight into how KSHV infection influences the humoral immune response affecting KSHV-related diseases.

## Materials and Methods

### Cell Culture

iSLK, iSLK.219, and iSLK.BAC16 cells (Brulois et al., 2012) were cultured in DMEM with 10% FBS and selection antibiotics as described previously (Myoung and Ganem, 2011b). CH12F3-2 cells were cultured in RPMI 1640 medium supplemented with 10% FBS, 0.05% β-mercaptoethanol, and 5% NCTC 109 (Sigma-Aldrich).

### rKSHV.219 Production

iSLK.219 cells were reactivated with 1 mM sodium butyrate and 1 μg/mL doxycycline without antibiotics at 60% confluence. The supernatant was collected after 4 days, centrifuged at 4000 rpm for 10 min, and filtered through a 0.45 μM PES membrane. This supernatant was spun at 27,000 × *g* for 90 min at 4°C. After ultracentrifugation, the viral pellet was resuspended in 1X PBS and stored in the -80°C for future use.

### rKSHV.219 UV-Irradiation

Purified rKSHV.219 was UV-irradiated with 150,000 J/cm^2^ of energy for two rounds. UV-inactivation was verified by spinoculating AD293 cells at 700 rpm for 60 min at 37°C with 8 μg/ul polybrene, and infection media was replaced with complete DMEM. At 48 h post-infection, FACS (LSRII) analysis ensured no GFP-positive cells. rKSHV.219 and UV-rKSHV.219 from the same viral stock were used within the same experiment.

### KSHV Infection

All infections were done in the presence of 5 μg/mL protamine sulfate. For CH12F3-2 infection via co-culture with iSLK.219 cells, 25 × 10^4^ iSLK.219 cells were reactivated for 24 h with 1 μg/mL doxycycline. After 24 h, co-culture was started by introducing 1:1 ratio of CH12F3-2 cells to the iSLK cells. After 24 h, αCD40, IL-4, and TGFβ (+CIT), were added to stimulate switching in CH12F3-2 cells and left for 48 h more before evaluating infection. For CH12F3-2 cells infected directly with purified rKSHV.219, 25 × 10^4^ CH12F3-2 cells were cultured with an MOI = 5 in 12 × 75 mm FACS tubes in 400 μL of serum-free RPMI at 37°C for at least 5 h. A final concentration of 10% FBS was added to the tubes and left overnight at 37°C in 1mL final volume. After 24 h, fresh CH12F3-2 media was added to the cells and they were transferred to a 6-well plate. Cells were maintained in inoculum until harvested. For primary naïve splenocytes infected with purified rKSHV.219, 1 × 10^6^ cells were cultured with an MOI of 1 or 5 in 12 × 75 mm FACS tubes in 400 μL of serum-free RPMI at 37°C for at least 5 h, or spun at 300 *g* for 30 min at 4°C and left at 37°C for 1 h. A final concentration of 10% FBS was added to the tubes, cells were transferred to a 6-well plate and LPS was added, and the culture was left overnight at 37°C in 2 mL final volume. The next day IL-4 was added to stimulate switching, fresh media was added as needed, and splenocytes were maintained in inoculum until used for experiments.

### Mice

Mice were housed under pathogen-free conditions. Animal studies were performed according to protocols approved by the Institutional Animal Care and Use Committee at the University of Miami. Splenocytes were removed from 8 to 10-week-old c57BL6/J male mice obtained from the Jackson Laboratory.

### CSR Induction

Mouse B cells were purified from freshly isolated splenocytes using anti-CD43 magnetic beads MACS CD43 depletion (Miltenyi Biotech) according to manufacturer’s protocol. Cells were stained with CFSE (Invitrogen) or eFlour670 (Thermo Fisher Scientific) and 5 × 10^5^ cells/mL were activated with 5 μg/mL LPS (Sigma) and 20 ng/mL murine IL-4 (Peprotech) (IgG1 switching), or 1 ng/mL TGF-β1 (IgG2b switching). Isotype switching was analyzed by FACS after staining cells with anti–IgG1 or IgG2b-biotin (BD Pharmingen), followed by PE-conjugated anti-biotin antibody. Splenocytes were cultured with or without purified rKSHV.219 (MOI = 1–5) with 5 μg/mL protamine sulfate and activated with the aforementioned cytokines. For CH12F3-2 experiments, cells were also preincubated with CFSE or eFluor 670 and 25 × 10^4^ cells/mL were activated with 1 ng/mL TGF-β1 (R&D Systems), 10 ng/mL recombinant murine IL-4 (PeproTech), and 1 μg/mL purified anti–mouse CD40 (BD Biosciences). Cells were stained with anti-IgA (SouthernBiotech) and analyzed by FACS with Accuri C6 Flow Cytometer (BD Biosciences).

### Lentiviral Infection

293TX cells were transfected at 70% confluence with pCMV delta 8.2, pCMV VSV-G, and the pSin, vFLIP, vGPCR, or vIL-6 plasmids from the Boshoff lentiviral library using Polyplus transfection reagent. Next day, the transfection media was replaced with fresh plating media and supernatant with virus was collected 48 h later. Virus was concentrated from the supernatant using LentiX concentrator (Clontech) according to the manufacturer’s protocol and resuspended in 1X PBS. Cells were infected adding 5 μg/ml of protamine sulfate to the culture media. Class-switching was induced in CH12F3-2 or mouse splenic B cells 24–48 h post-infection.

### qRT-PCR

To remove cell bound virus, all samples cultured with KSHV were washed 1X with PBS, trypsinized for 5 min at 37°C, and washed 2X in 1X PBS. RNA was harvested using RNeasy Kit (Qiagen) or Trizol with glycogen as a carrier as per the manufacturer’s instructions (Thermo Fisher Scientific). 500–1000 ng of RNA was used to make cDNA (Promega Improm-II). RT-qPCR was performed using an ABI Prism 7000 Sequence Detection System (Applied Biosystems) with SybrGreen PCR Master Mix (Quanta Biosciences). Analysis of qRT-PCR results was adapted from (Totonchy et al., 2018). As described above, total RNA was extracted from CH12F3-2 or primary splenic B cells that were mock infected or infected with rKSHV.219 (KSHV) and mRNA transcript expression for viral genes and rKSHV.219 infection markers (GFP, RFP) was validated using qRT-PCR for a 40-cycle reaction. To control for DNA contamination, there was no addition of Reverse Transcriptase (noRT) to at least 2 KSHV-infected cultures (biological replicates). Technical duplicates of the noRT Cq values (from all biological replicates) were used to calculate the mean noRT Cq value. Non-amplifying samples were set to Cq = 40 (not detected) for calculation purposes as all detected values were less than 40. The lowest Cq value from either the mean noRT control or mock-infected samples was selected to set the threshold for the limit of detection for each target gene. On the *Y*-axis of each graph, the Cq values are reversed where values ≥ 40 represent Cqs at or below the level of detection. The lower the Cq on the Y-axis (for example Cq = 27), the higher the gene expression in a sample for a target gene. Grey shading indicates values of the mock (black) or KSHV-infected (red) samples that fall at or below the threshold for the limit of detection. Green shading indicates values that are between 2.4 and 3.3 cycles less than the limit of detection (correlating to ∼5-10-fold increase in gene expression above the threshold detection limit). Red shading indicates values that are ≥3.3 cycles less than the limit of detection (correlating to ∼10-fold or greater increase in gene expression above the detection limit threshold). The cycle difference (ΔCq) is determined using the equation: (Cq for threshold of detection for target) – (Cq of KSHV-infected sample for target). The cycle difference (ΔCq) is converted to fold-change using the equation: 2ˆ(ΔCq). The mean and standard deviation of all samples for both the mock and KSHV groups are displayed on the graph. The following primer sets were used: AID, FW- GCCACCTTCGCAACAAGTCT, RV-CCGGGCACAGTCATAGCAC; Sμ, FW-TAGTAAGCGA GGCTCTAAAAAGCAC, RV-ACTCAGAGAAGCCCACCCAT; GAPDH, FW-TGAAGCAGGCATCTGAGGG, RV-CGAAGG TGGAAGAGTGGGAG. LANA, FW -CCTGGAAGTCCCAC AGTGTT, RV-AGACACAGGATGGGATGGAG, VIL-6, FW-, TGCTGGTTCAAGTTGTGGTC, RV-ATGCCGGTACGGTAA CAGAG, K8.1, FW-CACCACAGAACTGACCGATG, RV-TGGCACACGGTTACTAGCAC, GFP, FW-ACGTAAACGGC CACAAGTTC, RV- AAGTCGTGCTGCTTCATGTG, RFP, FW-AGGAGGGCTGCTTCATCTAC, RV-TGGTCTTCTTTGCA TCACG.

### Western Blotting

Cells were washed with 1X PBS and resuspended in RIPA buffer with Halt protease inhibitor cocktail (Thermo Scientific). Lysates were centrifuged at 10,000 *g* for 10 min at 4°C, resuspended in Laemmli buffer (Bio-Rad) and β-mercaptoethanol, and boiled for 5 min. Protein was quantified using the BCA protein assay (Thermo Scientific Pierce) and 35 μg was run on SDS-PAGE gels (Bio-Rad) in 1X Tris-glycine-SDS buffer (Bio-Rad) at 120 V, and transferred using 1X Tris-glycine onto a.2 μM PVDF membrane for 1 h at 100 V. Membranes were blocked in 5% BSA (phospho antibodies) or 5% non-fat milk (other antibodies) with TBS-T. Membranes were incubated with primary antibody (pSTAT3, STAT3, pSTAT, STAT1, pSTAT6, STAT6 (Cell Signaling Technology, 1:1000), AID (Active Motif, 1:500), Actin (Sigma, 1:10,000)) and in secondary antibody (Anti-rabbit, anti-mouse, goat-anti-rat; 1:10,000) and developed with Super Signal West Pico ECL western blotting substrate (Pierce) per the manufacturer’s instructions. Membranes were stripped with Restore PLUS Stripping Buffer (Thermo Fisher Scientific) per manufacturer’s instructions. Images were quantified by densitometry with Image Studio Lite software (Li-cor).

### Statistical Analysis

*P*-values are from a two-tailed Student’s *t*-test, unless stated otherwise. Bars represent the standard deviation (SD) obtained from 3 independent samples. Experiments were performed at least 2–3 times.

## Results

### KSHV Infection Enhances CSR Efficiency in Mouse CH12F3-2 Cells

**FIGURE 1:**
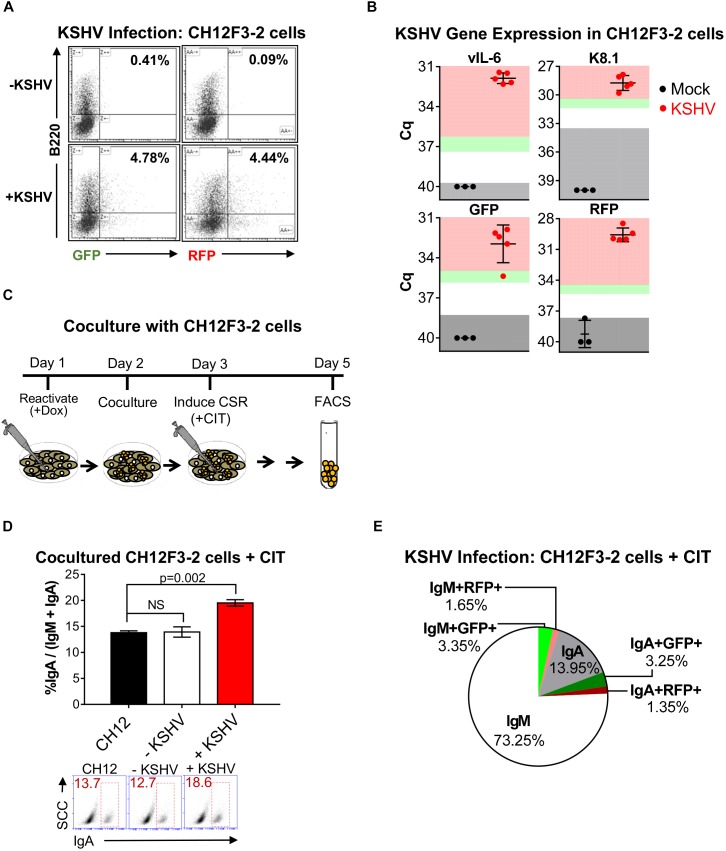
Kaposi’s sarcoma-associated herpesvirus infection enhances CSR efficiency in mouse CH12F3-2 cells. **(A)** Representative FACS plots showing the percentage of CH12F3-2 cells expressing GFP^+^B220^+^ and RFP^+^B220^+^ after 5 days of co-culture with lytically reactivated rKSHV.219-negative iSLK (–KSHV) and rKSHV.219-positive iSLK.219 (+KSHV) cells. Cytokines were not added to these cultures. Only single GFP^+^B220^+^ and RFP^+^B220^+^ cells were analyzed (doublets excluded based on FSC-A vs. FSC-W and SSC-A vs. SSC-W). **(B)** Three days post-infection, qRT-PCR was used to validate mRNA transcript expression for viral genes and rKSHV.219 infection markers from CH12F3-2 cell cultures that were mock-infected or infected with rKSHV.219 (KSHV). Gray shading indicates values of the mock (black) or KSHV-infected (red) samples that fall at or below the threshold for the limit of detection (TLD). Green and red shading indicate values corresponding to ∼5–10-fold increase, and ≥10-fold increase, in gene expression above the TLD, respectively. The Cq value for the TLD for each target was: 39.71 for vIL-6, 33.60 for K8.1, 38.31 for GFP, and 37.69 for RFP. Data is representative of Cq values from mock (*n* = 3) and KSHV-infected samples from two different experiments. A two-tailed Student’s *t*-test revealed a significant difference between Cq values for mock-infected and KSHV-infected samples, vIL-6, K8.1, RFP *p* = 0.0001, GFPp = 0.0002. **(C)** Schematic diagram depicting the co-culture strategy used to examine the effect of KSHV infection on CSR in CH12F3-2 cells. Day 1: 25 × 10^4^ iSLK (-KSHV) or iSLK.219 (+KSHV) cells were reactivated for 24 h with 1 μg/mL doxycycline. Day 2: A co-culture was started by introducing 25 × 10^4^ CH12F3-2 cells to the iSLK or iSLK.219 cells. Day 3: After 24 h of co-culture, cytokines, αCD40, IL-4, and TGFβ (+CIT), were added to stimulate CH12F3-2 cells for CSR from IgM to IgA. Day 5: After 48 h of stimulation, surface IgM and/ or IgA expression was assessed via FACS**. (D)** A representative experiment quantifying CSR from IgM to IgA at 48 h post-cytokine stimulation (+CIT) of CH12F3-2 (CH12) cells co-cultured with non-infected iSLK (–KSHV) cells or infected iSLK.219 (+KSHV) cells. Values are shown as the total percentage of IgA^+^ cells out of the sum of the total IgA^+^ and IgM^+^ population (% IgA/IgA + IgM). Below, A representative FACS plot displaying the percentage of IgA^+^ CH12F3-2 cells (inside red rectangle) **(E)** Pie chart displaying FACS analysis of CH12F3-2 cells co-cultured with iSLK.219 cells (+KSHV) from the experiment shown in **(D)** that were uninfected (IgM+ and IgA+), or resulted in a KSHV infection (IgM+GFP+, IgA+GFP+, IgM+RFP+, and IgA+RFP+). The 3.2% that is missing from the pie chart represents the population of cells that were likely iSLK cells (IgM-IgA-B220-).

It has been previously shown that KSHV infection upregulates AID expression in human tonsillar B cells (Bekerman et al., 2013). As AID induces CSR, we investigated the functional consequence of KSHV infection on CSR. We initiated our studies using CH12F3-2 cells, a unique mouse lymphoma system that undergoes class-switching from IgM to IgA in the presence of αCD40, IL-4, and TGFβ (CIT) (Nakamura et al., 1996; Cortizas et al., 2013). Although it is a murine model, it is currently the only reliable system to study the mechanisms of CSR *in vitro* (Stavnezer and Schrader, 2014). Recent studies demonstrated increased KSHV infection of B cells based on a mechanism of cell-contact by co-culturing them with lytically reactivated iSLK.219 cells (Myoung and Ganem, 2011c; Bekerman et al., 2013). In this system, iSLK.219 cells are infected with a recombinant KSHV construct, rKSHV.219, which expresses GFP under the elongation factor-1 (EF-1) promoter upon latency establishment, and expresses RFP under the polyadenylated nuclear (PAN) promoter when the virus is reactivated with doxycycline (Myoung and Ganem, 2011b). Hence, to establish infection efficiency of CH12F3-2 cells by KSHV, iSLK (KSHV-negative) or iSLK.219 (KSHV-positive) cells were reactivated for 24 h with doxycycline before the addition of CH12F3-2 cells. Five days post co-culture, CH12F3-2 cells were stained with anti-B220 (a B cell marker), to distinguish GFP or RFP-positive CH12F3-2 from iSLK.219 cells, and assessed for endogenous GFP and RFP expression using fluorescence-activated cell sorting (FACS). After 5 days of co-culture, approximately 4% of the CH12F3-2 cells expressed GFP or RFP (+KSHV, Figure 1A). To document and characterize the infection by KSHV of CH12F3 cells, we directly infected the CH12F3-2 cells with purified rKSHV.219. We validated expression of two KSHV lytic viral genes, vIL-6 and K8.1, in CH12F3-2 cells by qRT-PCR. The transcript level of both genes was greater than 10-fold the limit of detection (Figure 1B). The gene expression of rKSHV.219 infection markers, GFP and RFP, also showed more than a 10-fold difference over the limit of detection (Figure 1B), corroborating with the FACS data (Figure 1A), and further providing evidence of infection. While we observed GFP expression via FACS and qRT-PCR, LANA expression was negligible (data not shown), indicating a lytic infection. These data support evidence that KSHV produced from lytically reactivated iSLK.219 cells and purified rKSHV.219 results in infection of CH12F3-2 cells.

Next, we determined whether KSHV infection affected CSR in CH12F3-2 cells. For this objective, iSLK (-KSHV) and iSLK.219 (+KSHV) cells were reactivated for 24 h with doxycycline before introducing CH12F3-2 (CH12) cells to the culture (Figure 1C). After 24 h of co-culture, cytokines (CIT) were added, and 48 h later surface IgA expression was assessed via FACS (Figure 1C). CH12F3-2 cells were stained with both IgM and IgA to ensure that only CH12F3-2 cells were analyzed. As observed in Figure 1D, levels of class-switching were significantly elevated in CH12F3-2 cells co-cultured with reactivated cells (+KSHV, 24.8%) compared to CH12F3-2 cells that were cultured alone (CH12, 16.8%), or with uninfected cells (-KSHV, 15.7%) (Figure 1D). Furthermore, we consistently observed that the increase in CSR efficiency was always greater than the efficiency of KSHV-infected cells. Thus, a small population of cells infected with KSHV is mediating the increase in CSR in both the uninfected (GFP^-^, RFP^-^) and infected (GFP^+^, RFP^+^) cell population, suggesting that the effect of KSHV on CSR could have a paracrine component (Figure 1E). These data show that KSHV-infection enhances CSR in CH12F3-2 cells.

### KSHV Enhances Class-Switching Efficiency in Primary Mouse B Cells

**FIGURE 2:**
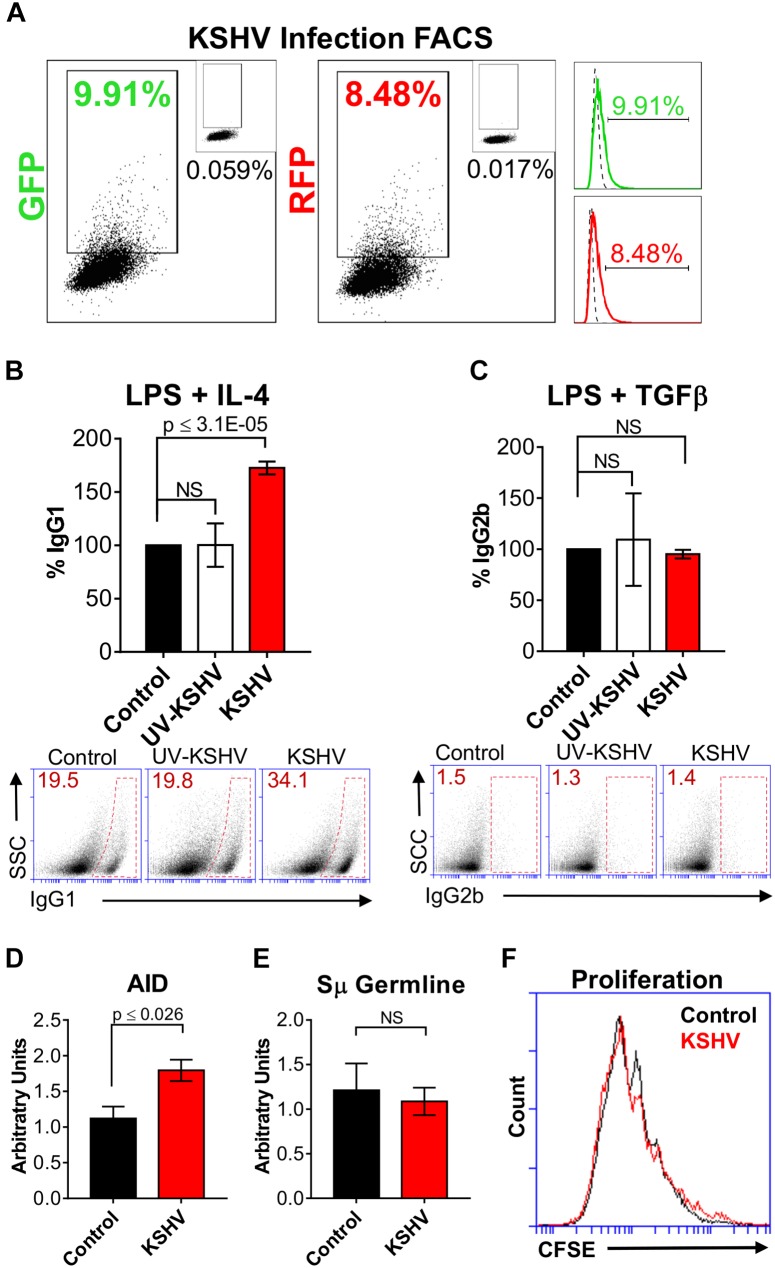
Kaposi’s sarcoma-associated herpesvirus enhances class-switching efficiency in primary mouse B cells. **(A)** Representative FACS plots of LPS/ IL-4 stimulated splenocytes showing the percentage cells expressing GFP^+^ and RFP^+^ (dot plot and histogram) at 48 h post-infection. The top-right inset of each dot plot shows GFP or RFP values for the mock-infected control. Histograms depict the dot plot data, where the dotted line represents the mock-infected control and the solid green or red line represents the shift in GFP or RFP expression, respectively. **(B)** 5 × 10^5^ cell/mL of naïve mouse splenic B cells were cultured with LPS (control) and purified rKSHV.219 (KSHV) or UV-irradiated rKSHV.219 (UV-KSHV). After 24 h, IL-4 was added to the cultures to stimulate IgG1-switching. Surface IgG1 expression was measured by FACS after 4 days of IL-4 stimulation. Values on the graph show the percentage of IgG1^+^ (% IgG1) cells relative to the control. Below, **(A)** representative FACS plot displays the raw values splenocytes that class-switched to IgG1^+^ (inside red polygon). **(C)** 5 × 10^5^ cell/mL of naïve mouse splenic B cells were cultured with LPS (control) and/ or purified rKSHV.219 (KSHV) or UV-irradiated rKSHV.219 (UV-KSHV). After 24 h, TGFβ was added to the cultures to stimulate IgG2b-switching. Surface IgG2b expression was measured by FACS after 4 days of stimulation. Values on the graph show the percentage of IgG2b^+^ (% IgG2b) cells relative to the control. Below, **(A)** representative FACS plot displays the raw values of splenocytes that class-switched to IgG2b^+^ (inside red rectangle). **(D)** AID and **(E)** Sμ germline mRNA transcripts were assessed using qPCR from splenic B cells cultured with LPS/IL-4 (control) or LPS/IL-4 /rKSHV.219 (KSHV). Values on the graph are calculated relative to the control and are depicted as arbitrary units. **(F)** Representative FACS plot evaluating cell proliferation using a CFSE dilution assay in splenic B cells cultured with LPS/IL-4 (control) or LPS/IL-4/rKSHV.219 (KSHV).

We chose to continue investigating the effect of KSHV on CSR using primary mouse splenic B cells because the parameters of class-switching are well-defined and established within this *in vitro* model in contrast to human cells (Stavnezer and Schrader, 2014). Initially, to determine the infection efficiency of KSHV in splenocytes, resting B cells were purified, cultured with rKSHV.219 (KSHV) and LPS, and endogenous GFP and RFP expression was assessed using FACS. After 48 h of infection, approximately 10% of the splenocyte population was GFP-positive and 8.5% was RFP-positive (Figure 2A). Like the CH12F3-2 cells (Figure 1A), KSHV-cultured splenocytes expressed both GFP and RFP, although a greater percentage of the splenocyte population showed fluorescent expression (Figure 2A) compared to the CH12F3-2 cells (Figure 1A). As this indicated that splenocytes could be infected with KSHV, resting B cells were purified, concomitantly cultured with LPS and rKSHV.219 (KSHV) or UV-irradiated KSHV (UV-KSHV), and after 24 h, stimulated with IL-4 (for switching to IgG1) or TGFβ-1 (for switching to IgG2b). We chose IL-4 and TGFβ because of the effect on CSR efficiency that we observed during KSHV infection in the CH12F3-2 system following stimulation with those cytokines (Figure 1D). After 4 days of cytokine exposure, surface IgG1 and IgG2b expression was assessed via FACS (Figures 2B,C). LPS/IL-4-activated splenic B cells cultured with KSHV exhibited a ∼70% increase in class-switching to IgG1 compared to B cells cultured without virus or with UV-KSHV (Figure 2B), indicating that replication-competent virus, not antigen exposure, enhanced CSR. In general, the efficiency of CSR to IgG1 increased more than 50% when exposed to KSHV. In contrast, B cells cultured with KSHV and stimulated with LPS/TGFβ exhibited no difference in the efficiency of class-switching to IgG2b when compared to control cells (Figure 2C). Noteworthy, KSHV always required the addition of cytokines to enhance class-switching efficiency in B cells. Splenic B cells or CH12F3-2 cells cultured with KSHV alone did not undergo switching. Splenocytes cultured with LPS and KSHV did not show enhanced CSR to IgG3 compared to splenocytes cultured with LPS alone (data not shown). KSHV-infection only enhanced the efficiency of CSR in splenocytes cultured with LPS/IL-4 (Figure 2B) and in CH12F3-2 cells cultured with CIT (Figure 1D). This shows that KSHV can enhance, but not promote CSR. These data indicate that KSHV enhances the efficiency of CSR to some isotypes in the presence of certain cytokines, and that viral infection mediates this effect.

Class-switching can be affected by differences in AID expression, germline transcription, or cell proliferation. To further determine the mechanism whereby KSHV enhanced CSR efficiency in B cells, we evaluated AID and germline transcript mRNA levels (Figures 2D,E), and cell proliferation (via CFSE staining, Figure 2F). Control or KSHV-cultured splenocytes stimulated for class-switching to IgG1 with LPS and IL-4 exhibited no substantial difference in Sμ germline mRNA levels (Figure 2E) or cell proliferation (Figure 2F). However, we observed significantly higher levels of AID mRNA transcripts in B cells exposed to KSHV (Figure 2D). This coincided with a trend toward decreased microhomology use for the DNA end-joining step in host IgH Sμ-Sγ1 regions, and increased transition mutations (Supplementary Figure 1). These data suggest that the observed increase in CSR to IgG1 in KSHV-infected B cell cultures may be due to upregulation of AID.

### KSHV vIL-6 Increases the Efficiency of CSR

**FIGURE 3:**
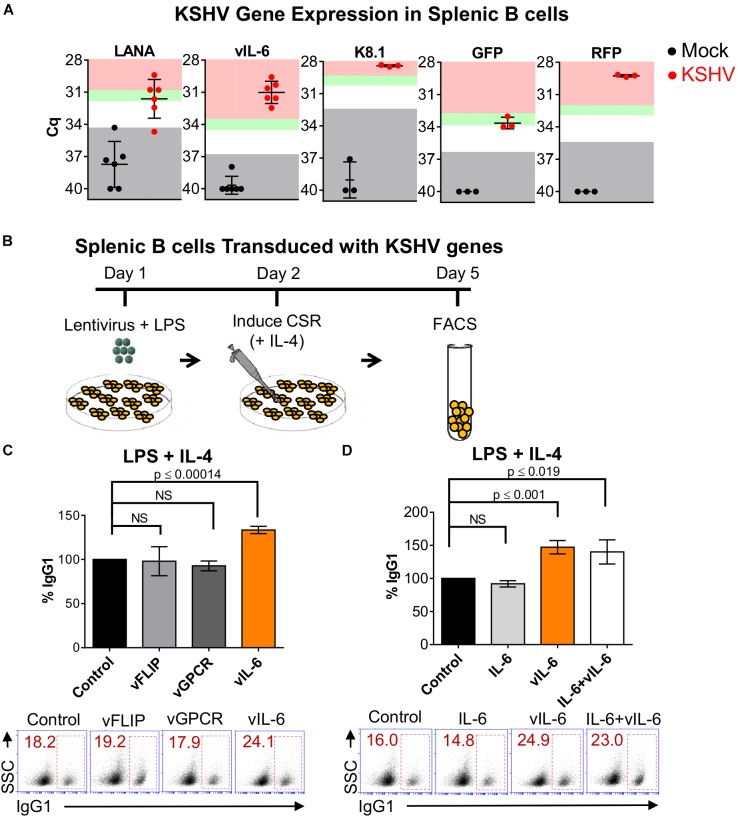
Kaposi’s sarcoma-associated herpesvirus vIL-6 increases the efficiency of CSR. **(A)** Four days post-infection, qRT-PCR was used to validate mRNA transcript expression for viral genes and rKSHV.219 infection markers from LPS/ IL-4-stimulated splenocyte cell cultures that were mock infected or infected with rKSHV.219 (KSHV). Gray shading indicates values of the mock (black) or KSHV-infected (red) samples that fall at or below the threshold for the limit of detection (TLD). Green and red shading indicate values corresponding to ∼5–10-fold increase, and ≥10-fold increase, in gene expression above the TLD, respectively. The Cq value for the TLD for each target was: 34.2 for LANA, 36.92 for vIL-6, 32.48 for K8.1, 36.28 for GFP, and 35.38 for RFP. Data is representative of Cq values from splenocyte cultures from 3 to 6 mice. A two-tailed Student’s *t*-test revealed a significant difference between Cq values for mock-infected and KSHV-infected samples, LANA *p* = 0.0003, vIL-6 *p* = 0.0001, K8.1 *p* = 0.0004, GFP *p* = 0.0013, RFP *p* = 0.0001. **(B)** Schematic diagram depicting the strategy used to determine how expression of lentiviral plasmids coding for different KSHV viral proteins affect the efficiency of CSR in naïve mouse splenic B cells. Day 1: 5 × 10^5^ cells/mL were transduced with KSHV lentiviral plasmids from a KSHV lentiviral library. Day 2: Twenty-four hours later, B cells were stimulated for switching with LPS and IL-4 to IgG1, Day 5: surface IgG1 expression was analyzed by FACS after 4 days of stimulation. **(C)** Top, efficiency of class switching to IgG1 in mouse splenic B cells transduced with the pSin empty plasmid (control), or KSHV genes (vFLIP, vGPCR, or vIL-6) and stimulated for 4 days with LPS and IL-4. Bottom, **(A)** representative FACS plot displaying the percentage of splenocytes that class-switched to IgG1^+^ (inside red rectangle) is shown. **(D)** Top, efficiency of class switching to IgG1 in mouse splenic B cells transduced with the pSin empty plasmid (control), or KSHV vIL-6 (vIL-6), or treated with exogenous murine IL-6 (IL-6), or concomitantly transduced with KSHV vIL-6 and treated with murine IL-6 (IL-6+vIL-6) and stimulated for 4 days with LPS and IL-4. Bottom, **(A)** representative FACS plot displaying the percentage of splenocytes that class-switched to IgG1^+^ (inside red rectangle) is shown. Values on the graphs in **(B,C)** show the percentage of IgG1^+^-switched cells (% IgG1) relative to the control.

In seeking for a viral-mediated mechanism for the enhanced CSR, we first assessed viral gene expression within the population of KSHV-infected LPS/IL-4 stimulated splenic B cells via qRT-PCR. We observed expression of the latent, LANA, and lytic, vIL-6 and K8.1, viral genes. LANA transcripts were more than 5-fold (except for one mouse), while vIL-6 and K8.1 levels were greater than 10-fold the limit of detection (Figure 3A). The mRNA transcripts of rKSHV.219 infection marker, GFP, was 3-to5-fold, and RFP, was higher than 10-fold the detection limit (Figure 3A), supporting the FACS data (Figure 2A). This indicates that there are both latently and lytically infected cells within the splenocyte population. These data substantiate that primary splenic B cells stimulated for switching to IgG1 can be infected with KSHV.

Due to evidence of KSHV gene expression within KSHV-infected cultures of CH12F3-2 cells (Figure 1B) and primary splenocytes (Figure 3A), we hypothesized that particular viral genes could be influencing class-switching. To elucidate the viral mechanism mediating increased CSR, we evaluated the ability of individual KSHV genes to modulate CSR efficiency. Class-switching is prompted by a combination of cytokines and T cell-dependent or T cell-independent signals which trigger JAK/ STAT and NF-κB pathways to induce AID expression (Xu et al., 2012). Thus, the latent viral FLICE-inhibitory protein (vFLIP), and the lytic viral G protein-coupled receptor (vGPCR) gene candidates were selected based on their ability to directly or indirectly activate NF-κB (Mesri et al., 2010). vFLIP was also chosen because it upregulated AID expression in primary human tonsillar B cells (Bekerman et al., 2013). Besides its capacity to signal through the JAK/STAT pathway (Mesri et al., 2010; Xu et al., 2012), vIL-6 was selected due to its paracrine signaling potential (Mesri et al., 2010) and consistent levels of gene expression within KSHV-infected cultures of CH12F3-2 cells (Figure 1B) and splenocytes (Figure 3A). Accordingly, mouse naïve splenic B cells were transduced with vFLIP, vGPCR, or vIL-6 from a KSHV lentiviral library (Vart et al., 2007), and activated with LPS (Figure 3B). Twenty-four hours post-lentiviral infection, IL-4 was added to the culture to induce class-switching to IgG1, and surface isotype expression was evaluated via FACS 4 days later (Figure 3B). We observed that vIL-6 significantly increased class-switching in mouse splenocytes (33%, *p* ≤ 0.00014), in contrast to both vFLIP and vGPCR which resulted in no significant change in switching to IgG1 compared to the control (Figure 3C).

Viral cytokines such as vIL-6 often exhibit gain-of-function properties. Several differences exist between vIL-6 and IL-6 in regards to signaling, subcellular localization, secretion, host-binding interactions, and endothelial cell movement (Wan et al., 1999; Nicholas, 2005; Hu and Nicholas, 2006; Chen et al., 2009; Chen et al., 2012, 2014; Cousins et al., 2014; Giffin et al., 2015). Therefore, we wanted to determine whether mIL-6 affected class-switching, and/or if it demonstrated synergism with its viral homolog (Suthaus et al., 2012). Thus, B cells were transduced with vIL-6 lentivirus, and/or incubated with recombinant mIL-6, and CSR to IgG1 was evaluated. While IL-4-stimulated B cells transduced with vIL-6 resulted in a 47% increase in class-switching to IgG1 (Figure 3C), cells treated with mIL-6 showed no difference to control cells (Figure 3D). Similarly, mIL-6 did not further influence CSR levels in stimulated splenocytes transduced with vIL-6, suggesting that this effect is specific to vIL-6 (Figure 3D). These results indicate that vIL-6 contributed to the enhanced levels of class-switching mediated by KSHV.

### vIL-6 Increases AID Expression in B Cells Stimulated for CSR

**FIGURE 4:**
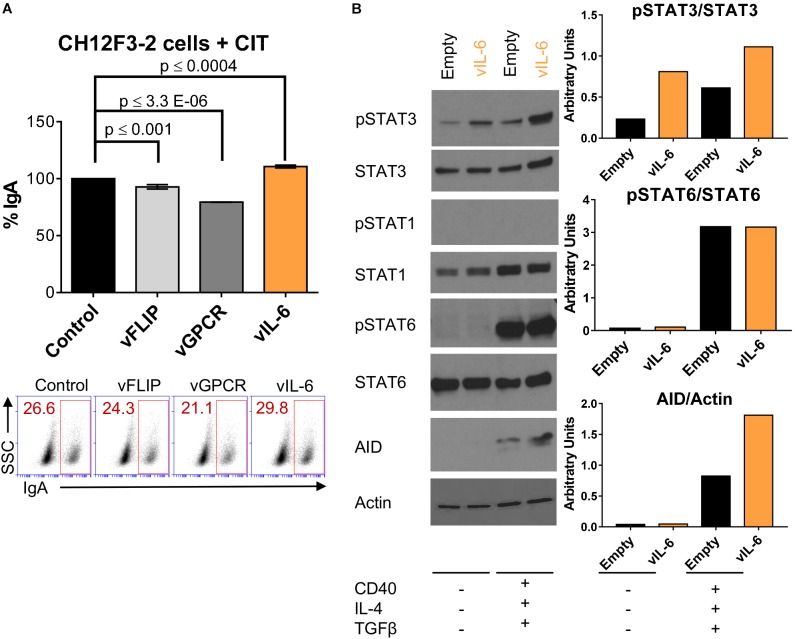
vIL-6 increases phosphorylation of STAT3 and AID expression in B cells stimulated for CSR. **(A)** A representative experiment quantifying CSR to IgA after 24 h of CIT stimulation (+CIT) in CH12F3-2 cells transduced with the pSin plasmid (control), and KSHV genes (vFLIP, vGPCR, and vIL-6). Values on the graph show the percentage of IgA^+^ cells (% IgA) relative to the control. Below, **(A)** representative FACS plot displaying the percentage of CH12F3-2 cells that switched to IgA (inside red rectangle). **(B)** Left, Western blot images of total extract from CH12F3-2 cells transduced with the pSin plasmid (Empty) or with KSHV vIL-6 (vIL-6) and stimulated for 24 h without (–) or with (+) αCD40, IL-4, and TGFβ to induce CSR to IgA. Right, Graphs showing quantification in arbitrary units of phospho-STAT3 relative to total STAT3 (pSTAT3/STAT3), phospho-STAT6 relative to total STAT6 (pSTAT6/STAT6) and AID relative to Actin (AID/Actin).

AID initiates CSR (Muramatsu et al., 2000) and elevated AID expression is associated with increased class-switching efficiency (Cortizas et al., 2013). As we observed significantly higher levels of AID transcripts (Figure 2D) and IgG1 isotype-switching in stimulated splenic B cell cultures infected with KSHV (Figure 2B), we explored if vIL-6 expression was able to upregulate AID expression in the context of our CH12F3-2 CSR system. Initially, CH12F3-2 B cells were transduced with vFLIP, vGPCR, and vIL-6 (Figure 4A) to determine their effect on CSR efficiency. Although the increase in the level of IgA-switching observed in CH12F3-2 B cells transduced with vIL-6 was lower than that of IgG1 observed with mouse splenic B cells (Figures 3C,D), vIL-6 expression showed a trend to augment switching to IgA (∼10%), which was not observed with vFLIP or vGPCR (Figure 4A).

Accordingly, CH12F3-2 cells were transduced with empty vector or vIL-6 and stimulated or not with cytokines (CIT) for 24 h to evaluate the consequence of vIL-6 expression on AID levels. Since vIL-6 could activate both STAT1 and STAT3 (Molden et al., 1997), we used the phosphorylation of these two transcription factors to assess vIL-6 downstream signaling. We observed that vIL-6 transduction in CH12F3-2 cells upregulated phospho-STAT3 in the absence of CIT, and that cytokine stimulation amplified this effect (Figure 4B and Supplementary Figure 3). However, while total STAT1 expression slightly increased with cytokine exposure (Figure 4B), phospho-STAT1 expression was undetected in either the empty vector or vIL-6 transduced cells (Figure 4B and Supplementary Figure 3). These data suggest that in this B cell system, vIL-6 activates STAT3, and not STAT1 phosphorylation. Since STAT6 is implicated in CSR and is upregulated in response to IL-4 signaling (Dedeoglu et al., 2004), we used STAT6 as a positive control for IL-4 stimulation and to further show the signaling specificity of vIL-6 in CH12F3-2 cells. Phospho-STAT6 was substantially upregulated when stimulated with cytokines as predicted, and there was no difference in expression between control and vIL-6 transduced cells (Figure 4B). As expected, AID was only expressed in the presence of cytokines and its levels increased with vIL-6 expression (Figure 4B and Supplementary Figure 3), suggesting that AID expression was driven by STAT6-activating cytokines and was further augmented by vIL-6 signaling (Figure 4B). These data indicate that vIL-6 enhances class-switching likely by upregulating AID expression in B cells.

## Discussion

While it is clear that KSHV influences the immune system, the mechanisms of how acute KSHV infection modulate adaptive immunity, particularly aspects of Ig diversification, are not fully understood. B lymphocytes are integral to an effective adaptive immune response. The effectiveness is achieved in part by the ability of antigen-activated mature B cells to undergo CSR, altering their constant region to produce isotype-switched antibodies with distinct effector functions. The current study shows that KSHV impacts Ig diversification by enhancing CSR. We demonstrate that KSHV-infection in combination with cytokines and inflammatory environments enhanced CSR and upregulated AID transcripts. Further, the KSHV-encoded lytic viral gene, vIL-6, contributed to elevated levels of CSR that correlated with increased AID expression. These data identify a potential novel function for vIL-6 as a contributing factor to KSHV-induced Ig CSR.

Our results are consistent with, and further expand on a study showing that AID mRNA levels increase in B cells infected with KSHV (Bekerman et al., 2013). We do this by showing that the functional consequence of increased AID transcripts is enhanced CSR, and by using single gene lentiviral transduction, identifying vIL-6 as one of the potential KSHV genes implicated in enhancing the efficiency of class-switching (Figures 3C,D) via increased AID expression (Figure 4B). Further, it was previously demonstrated that vFLIP induced AID transcripts in human tonsillar B cells (Bekerman et al., 2013). Yet, we found that transduction of vFLIP or vGPCR did not enhance CSR levels (Figure 4A) in the conditions of our murine assays. We acknowledge that utilizing murine models to investigate KSHV in regards to Ig diversification represents a limitation of our study, as previous studies used human tonsillar B cells (Bekerman et al., 2013; Totonchy et al., 2018). However, the systems we employed are the only well-characterized *in vitro* models currently used to evaluate CSR, due to the lack of available human systems (Stavnezer and Schrader, 2014).

AID expression and its consequential effects on CSR could depend on whether infection is latent or lytic as the type of infection determines the effect of viral genes on the host regulatory machinery. As shown in Figures 2A, 3A, we provide evidence of latently and lytically infected murine splenocytes. Although we detected mRNA transcripts and fluorescent GFP protein expression, our analysis revealed that KSHV-infection in CH12F3-2 cells is mostly lytic with negligible LANA detection, while splenocytes express both latent and lytic genes. Interestingly, our KSHV infection results are similar to other publications which not only show concomitant latently and lytically infected primary peripheral blood mononuclear and human tonsillar B cells, but also report patterns of LANA and K8.1 expression (Rappocciolo et al., 2008; Totonchy et al., 2018) that are comparable to those we observe in our mouse splenic B cell cultures. The congruency in *de novo* infection and viral gene expression among primary human and mouse B cells further supports employing mouse systems to study KSHV in the context of CSR.

Our system for *de novo* KSHV-infection of B cells *in vitro* yields lower percentages of infected cell populations, which are consistent with other published reports on infection of primary B cells (Myoung and Ganem, 2011a; Bekerman et al., 2013; Knowlton et al., 2014; Totonchy et al., 2018) (Figures 1A, 2A). We show that CSR is enhanced in splenocytes cultured only with replication-competent KSHV suggesting that infection is an important component for enhancing the efficiency of CSR. However, we found that the extent of class-switching that occurred in B cells stimulated for CSR was always greater than the percentage of cells expressing GFP or RFP (Figures 1A, 2A), implying that the increased class-switching levels were due to direct and paracrine effects. This paracrine mechanism is consistent with the possibility that lytically infected cells, which may be poised for cytopathic effects, would still be able to produce factors that may enhance CSR in a paracrine manner. Although it is uncertain whether KSHV seropositivity is due to a recent infection or viral reactivation, seroconversion is observed years before the onset of KS, suggesting that the appearance of immunoglobulins is due to repeated and subdued levels of viral lytic replication (Gao et al., 1996; Biggar et al., 2003) which can simulate recurrent episodes of acute infection. Our data supports this hypothesis as we observed elevated class-switching during KSHV reactivation (Figure 1D), upon low levels of *de novo* infection resulting in vIL-6 and K8.1 gene expression (Figures 1B, 3A), and with vIL-6 lentiviral transduction in B cells (Figures 3C,D), implying that KSHV replication and paracrine signaling may contribute to this process.

We show that vIL-6 enhanced CSR efficiency in primary B cells while the mouse cytokine did not have any effect (Figure 3D). These data support the idea that additional unidentified functional differences exist between the IL-6 host and viral homologues which may be explained by vIL-6’s ability to directly bind to gp130 and initiate signaling, localize to the ER, or its prolonged half-life kinetics (Nicholas et al., 1997; Wan et al., 1999; Nicholas, 2005; Hu and Nicholas, 2006; Chen et al., 2009). We are cognizant that lentiviral transduction of vIL-6 may produce levels of this viral cytokine that are much greater than those in KSHV-infected cells. However, we found that exogenously expressed vIL-6 transcript levels in CH12F3-2 cells were augmented more than100-fold relative to the uninfected control (Supplementary Figure 2), and that KSHV-infected splenocytes expressed vIL-6 more than 50-fold the limit of detection. Since the efficiency of vIL-6 lentiviral transduction is higher than KSHV infection, expression of vIL-6 at the single cell level could be comparable.

We show that vIL-6 substantially upregulated STAT3 phosphorylation in CH12F3-2 cells alone, and when stimulated with αCD40, IL-4 and TGFβ for class-switching, which coincided with elevated AID expression (Figure 4). However, vIL-6 did not affect STAT1 or STAT6 phosphorylation (Figure 4). CD40 ligation (Hanissian and Geha, 1997) and IL-4 stimulation (Rolling et al., 1996) can trigger STAT3 phosphorylation. IL-4 stimulation also induces STAT6 phosphorylation and nuclear translocation (Linehan et al., 1998) whereby STAT6 binds to a 5′ upstream promoter region on the AID gene to induce AID expression (Dedeoglu et al., 2004). STAT3 has been implicated in gamma herpes virus latency establishment in B cells (Reddy et al., 2016), plasma cell differentiation (Fornek et al., 2006) and IgG1-switching in a murine model of lupus pathogenesis (Ding et al., 2016). Further experimentation is necessary to fully understand the mechanism of vIL-6-mediated AID upregulation and enhanced CSR, and to determine which transcription factors, including STAT3, are involved in this process.

We observed that murine splenocytes exposed to KSHV only augmented class-switching efficiency to IgG1 when stimulated with IL-4 (Figure 2A), an immunomodulatory cytokine secreted by Th2 and mast cells (Husain et al., 1997). Immune dysfunction and cytokine dysregulation are implicated in KSHV-associated disorders. Cytokines direct CSR by determining the isotype of an antibody. *In vitro*, KSHV infects and replicates in human B cells activated with IL-4 (Hensler et al., 2014). Patient-derived AIDS-KS cells can produce IL-4 (Sirianni et al., 1998) and express surface IL-4R (Husain et al., 1997, 1999). KSHV is also hypothesized to favor a Th2-mediated response due to inflammation generated from hyperactivation of the humoral immune system, which is paralleled with a diminished Th1-mediated antiviral response (Douglas et al., 2010). Additionally, IL-4 prompts isotype-switching to IgG1 and IgE, in mice, but in humans to IgE or IgG4 (Mestas and Hughes, 2004). While the IL-4-stimulated isotype effector function in the mouse and human cannot be directly correlated, elevated levels of IgE have shown to be a prognostic marker of poor outcome for KSHV non-Hodgkin lymphomas (Uldrick et al., 2014) and associated with the extent of KS lesion development (Barbachano-Guerrero et al., 2017).

Kaposi’s sarcoma-associated herpesvirus is detected with concomitant infection of HIV and EBV, and causes several lymphoproliferative disorders. MCD arises from naïve B cells infected with KSHV (Totonchy, 2017), while PEL and GLD originate from KSHV/EBV-infected B cells of a GC or post-GC origin that have undergone class-switching (Totonchy, 2017). Although MCD or PEL B cells usually express IgM, both IgM and IgA isotypes are documented with GLD (Du et al., 2002; Bhavsar et al., 2017). Typically, CSR transpires in GC B cells. However, class-switching can also occur via an extrafollicular maturation pathway which ensues via a modified, or independent of a GC reaction to produce IgM and IgG immunoglobulins (Totonchy, 2017). It has been suggested that MCD lymphocytes display characteristics of this pathway (Totonchy, 2017). It was also recently shown that human tonsillar B cells infected with KSHV alter Ig light chain specificity (Totonchy et al., 2018). Further, predominant characteristics of plasma cell-type MCD occurred in transgenic mice that constitutively expressed vIL-6, particularly plasmacytosis and IgG hypergammaglobulinemia (Suthaus et al., 2012), suggesting that vIL-6 may play a role in enhancing serum Ig levels. These data points to/suggest a role for isotype–switching in settings of co-infection, but its contribution to the development of KSHV-related lymphoproliferative diseases needs to be further investigated.

Furthermore, studies focused on KS in KSHV/HIV infected populations within the United States and Zambia reveal evidence for isotype-switched antibodies with inconsistent effector function. Few children produce neutralizing antibodies (NAb) after primary infection with KSHV, although seroconversion is frequent and total KSHV antibodies increase (Olp et al., 2016). KSHV-Nab are both reduced (Kimball et al., 2004) and elevated (Kumar et al., 2013) in KS-positive adults. Additionally, elevated levels of IgA (Taylor et al., 1986; Rozenbaum et al., 1990; Spano et al., 2000) and IgG (Taylor et al., 1986; Spano et al., 2000) are associated with poor prognosis and KS disease progression, as well as with KS regression upon immune restoration with HAART (Mbopi-Keou et al., 2004). Although we did not examine the effector function of the KSHV-exposed class-switched B cells, our study similarly shows that KSHV could augment switching to IgG1 and IgA. Total serum IgG also increases upon infection with the murine equivalent to KSHV, MHV68, in mice (Stevenson and Doherty, 1998). The aforementioned findings indicate that within a functional immune system, isotype-switched antibodies correlate with KS regression, but KSHV-specific antibodies which lack neutralizing capabilities are also produced. In conjunction with immunosuppression, cytokine dysregulation, and other factors, class-switching to certain isotypes is associated with KS progression.

Our data showing the ability of KSHV to enhance the efficiency of class switching may provide a mechanistic explanation on how acute KSHV infection or recurrent lytic replication, in the context of certain cytokine and inflammatory environments, could affect humoral immunity in KSHV-associated diseases. The capability of vIL-6 to enhance CSR adds a new and intriguing biological capability for this pathogenic viral gene. Further research is warranted to elucidate the potential role of this viral protein in its ability to enhance class-switch recombination and its contribution to KSHV pathogenesis.

## Data Are Available on Request

The raw data supporting the conclusions of this manuscript will be made available by the authors, without undue reservation, to any qualified researcher.

## Author Contributions

RV and EM contributed conception of the study. All authors contributed to design of experiments. SR, GS, and RV performed the experiments, analyzed, and interpreted the data. SR wrote the draft of the manuscript and performed the statistical analysis. All authors contributed to manuscript revision, read and approved the submitted version.

## Conflict of Interest Statement

The authors declare that the research was conducted in the absence of any commercial or financial relationships that could be construed as a potential conflict of interest.
