# Supplementary material for: Kaposi’s Sarcoma-Associated Herpesvirus-Encoded Viral IL-6 (vIL-6) Enhances Immunoglobulin Class-Switch Recombination
Source: Front Microbiol. 2018 Dec 18;9:3119. doi: 10.3389/fmicb.2018.03119 (PMC6305588; doi:10.3389/fmicb.2018.03119)
Supplement: Supplementary file 1 [file Data_Sheet_1.pdf]

Supplementary Material

Kaposi's Sarcoma-Associated Herpesvirus-Encoded Viral IL-6 (vIL-6) Enhances Immunoglobulin Class-Switch Recombination

Santas A. Rosario, Gabriel E. Santiago, Enrique A. Mesri*, and Ramiro E. Verdun*
***Correspondence:** Corresponding Author: emesri@med.maimi.edu, rverdun@med.miami.edu

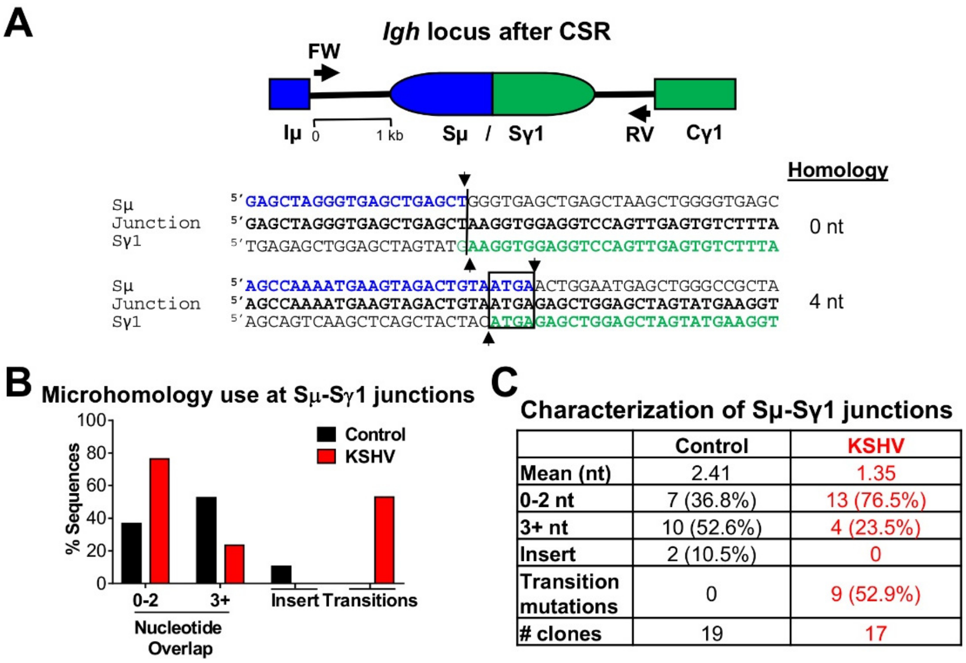

	Control	KSHV
Mean (nt)	2.41	1.35
0-2 nt	7 (36.8%)	13 (76.5%)
3+ nt	10 (52.6%)	4 (23.5%)
Insert	2 (10.5%)	0
Transition mutations	0	9 (52.9%)
# clones	19	17

Supplementary Figure 1. KSHV-exposed splenocytes show a trend to decrease microhomology use at Sμ-Sγ1 junctions compared to control cells **(A)** Top, A schematic diagram of a Sμ-Sγ1 switch junction. Arrows show the position of the forward (FW) and reverse (RV) primers used for the amplification of the switch junctions. Bottom, Examples of alignment of Sμ-Sγ1 junctions (bold black) with Sμ overlap (blue) and Sγ1 overlap (green) germline sequences. Arrowheads indicate breakpoints in Sμ and Sγ1, where the vertical line shows no homology (0 nt overlap), and the box highlights homology (4 nt overlap) at the junction. **(B)** Percentage of Sμ-Sγ1 junctions with the indicated length of microhomology which is identified as the largest matches to the germline sequence in primary splenic B cells stimulated for switching with LPS/IL-4 (control) or LPS/IL-4/rKSHV.219 (KSHV). Graphs displaying the percentage of sequences exhibiting nucleotide overlap, insertions, and transition mutations from control and KSHV clones. (**C**) Table comparing microhomology use at Sμ-Sγ1 junctions in LPS/IL4-stimulated mouse splenic B cells cultured without rKSHV.219 (control) or with rKSHV.219 (KSHV). Two-tailed Fisher exact test was used for p-value test, p=0.079

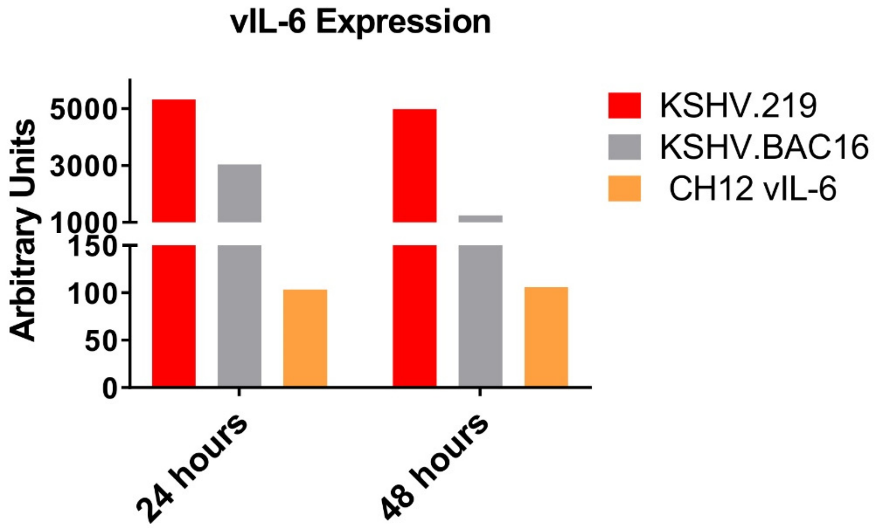

Supplementary Figure 2. Comparison of vIL-6 transcript levels in KSHV-infected and vIL-6 lentivirally transduced cells. RNA was harvested from rKSHV.219-infected, iSLK.219 (red), and KSHV BAC16-infected, iSLK.BAC16 (gray) cell lines lytically reactivated with doxycycline for 24 and 48-hours, and CH12F3-2 cells (orange) lentivirally transduced with vIL-6 for 24 and 48 hours. Graph displays values of fold-change of vIL-6 gene expression of KSHV-infected or vIL-6 transduced cells relative to uninfected control.

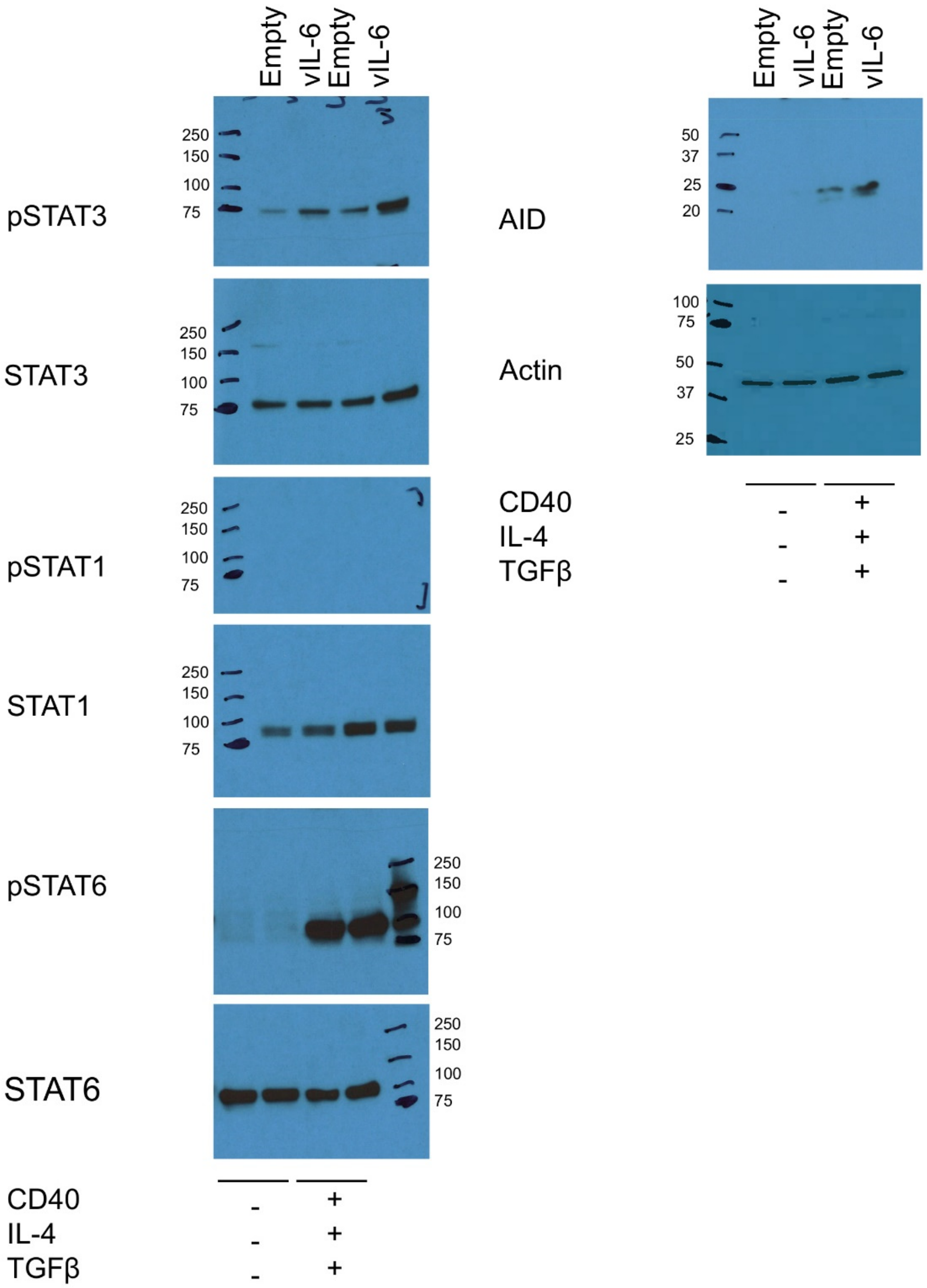

Supplementary Figure 3. Full image of the cropped western blot images shown in Figure 4 for CH12F3-2 cells transduced with the pSin plasmid (Empty) or with KSHV vIL-6 (vIL-6) and stimulated for 24 hours without (-) or with (+) αCD40, IL-4, and TGFβ to induce CSR to IgA. Numbers to the left or the right of the blot indicate the ladder in kilodaltons. One membrane was cut into 2-3 sections (~250-75 kDa, 50-10 kDa or 100-25 kDa) prior to incubation with antibodies in order to probe for multiple proteins using a single membrane.
